# Minimally Invasive Percutaneous Techniques for the Relief of Pain in Lumbar Disc Disease

**DOI:** 10.5812/kowsar.22287523.2102

**Published:** 2011-09-26

**Authors:** Tariq Sinan, Mehraj Sheikh

**Affiliations:** 1 Department of Radiology, Kuwait University Health Sciences Center, Jabriya, Kuwait; 2 Department of Radiology, Kuwait University and Mubarak Al-Kabeer Teaching Hospital, Jabriya, Kuwait

**Keywords:** Pain, Back pain, Disk prolapse

*Dear Editor*,

Alleviation of back pain and/or any disability due to pain in patients with lumbar disc disease undoubtedly gives a sense of relief and well being. The use of noninvasive or minimally invasive interventional procedures for this condition is basically aimed at limiting pain and disability. A number of such minimally invasive percutaneous techniques for the treatment of lumbar disc disease have been developed over the years, with the common tenet being the ability to act directly on the disc content without violating the spinal channel or increasing subsequent risk of scarring.

Electrosurgical technology has been developed for use in the spine and possesses few, if any of the drawbacks of the other percutaneous disc decompression techniques. These are usually plasma-mediated radiofrequency-based excision and allow precise etching of tissue. When used for tissue ablation (excision) in the disc, these procedures excise target material without gross thermal or structural damage to adjacent tissue (1).

We have studied patients using the nucleoplasty procedure developed by Arthro Care Corporation (Sunnyvale, CA), which is performed using a plasma-mediated radiofrequency-based device. This procedure uses radiofrequency to excite the electrolytes in a conductive medium, such as saline solution, to create precisely focused plasma. The energized particles in the plasma have sufficient energy to break molecular bonds, excising or dissolving soft tissue at relatively low temperatures (2-4). In our study (5) on 83 patients the majority of patients reported substantial pain relief within one month following the nucleoplasty discectomy, with the majority of them reporting minimal or no pain or disability and satisfaction with results after one year. Our experience revealed that nucleoplasty discectomy is a suitable, minimally invasive technique that could be considered a valid alternative to surgery when evaluating possible treatment options in patients affected by contained lumbar disc herniation. It appears particularly indicated for patients that have exhausted other conservative treatment modalities but have not yet reached clear-cut indications for open surgical treatment and in such patients it could be safe to give it a trial.

Two main mechanisms are reported as possibly being the basis of the effect of percutaneous disc decompression. Mechanical decompression of the nucleus pulpous with partial emptying of the disc space with consequent pressure lowering inside the disc space is considered as a mechanical explanation of the procedure (6). The other mechanism that has been hypothesized is a chemical disruption of degenerative metabolic processes inside the disc space which may induce changes in disc metabolism related to inflammation and/or initiate an autoimmune response in surrounding tissue to affect pain symptomatology (7-9). Based on these hypothesis it is postulated that nucleoplasty can be performed on patients with non-contained disc prolapse as well and that too at higher energy levels, unlike that of our study where it was done on selected patients with contained disc prolapse.

Farzanegan et al. (10) in a study published in this journal showed that pain relief after laminectomy, which is a major surgical procedure, for lumbar disc protrusion resulted in improvements in the mental depression that was associated with pain. The main difference between their study and ours was our study advocates the use of nucleoplasty, a non-invasive procedure, for pain relief. In summary, our studies, in conjunction to the study reported in this journal lend credence to the fact that pain relief, which is essential to the physical and mental well being of patients, should be acquired at least initially by non-invasive or minimally invasive procedures, and therefore we recommend nucleoplasty as an initial methodology for alleviation of back pain.
